# Crystal structure of a tetranuclear Cu^II^ complex with an *O*,*N*,*N*′-donor Schiff base ligand: hexa-μ_2_-acetato-bis­(2-{[(2,2,6,6-tetra­methyl­piperidin-4-yl)imino]­meth­yl}phenolato-κ^3^ *O*,*N*,*N*′)tetra­copper(II)

**DOI:** 10.1107/S2056989016005041

**Published:** 2016-03-31

**Authors:** Guohui Huang, Xiaoxuan Liu

**Affiliations:** a School of Materials and Energy, Guangdong University of Technology, No. 100 Waihuan Xi Road, Guangzhou, Guangdong 510006, People’s Republic of China

**Keywords:** crystal structure, Schiff base ligand, boat conformation, piperidines, copper(II) complex

## Abstract

The title compound, [Cu_4_(CH_3_COO)_6_(C_16_H_23_N_2_O)_2_], lies across a twofold rotation axis. The asymmetric unit contains two independent Cu^II^ ions. The symmetry-unique terminal Cu^II^ ion is *O*,*N*,*N*′-coordinated by a 2-{[(2,2,6,6-tetra­methyl­piperidin-4-yl)imino]­meth­yl}phenolate ligand and an O atom from an acetate group in a slightly distorted square-planar coordination environment. The symmetry-unique central Cu^II^ ion is coordinated by a different O atom from the same acetate group and by four bridging acetate ligands, which connect the asymmetric unit into a dimeric complex and form a distorted square-pyramidal coordination environment. Within the complex there are two symmetry-equivalent intra­molecular N—H⋯O hydrogen bonds. In the crystal, weak C—H⋯O hydrogen bonds link the complex mol­ecules, forming a three-dimensional network.

## Abstract 2

In the title compound, the symmetry-unique terminal Cu^II^ ion is *O*,*N*,*N*′-coordinated by a 2-{[(2,2,6,6-tetra­methyl­piperidin-4-yl)imino]­meth­yl}phenolate ligand and an O atom from an acetate group in a slightly distorted tetra­hedral coordination environment. The symmetry-unique central Cu^II^ ion is coordinated by a different O atom of the same acetate group and by four bridging acetate ligands, which connect the asymmetric unit into a dimeric complex and form a slightly distorted square-pyramidal coordination environment.

## Chemical context   

The chemistry of metal complexes with Schiff base ligands and their applications has attracted considerable attention, mainly due to their preparative accessibility, structural variability, magnetic properties and biological properties (Karahan *et al.*, 2015 ▸). The design of suitable building blocks and the utilization of coordinate bonds and non-covalent inter­actions to generate self-assemblies of various dimensions having aesthetic beauty and properties for possible use as functional materials are the major objectives in supra­molecular chemistry and crystal engineering (Sasmal *et al.*, 2011 ▸). Within this context, we report herein the crystal structure of the title complex.
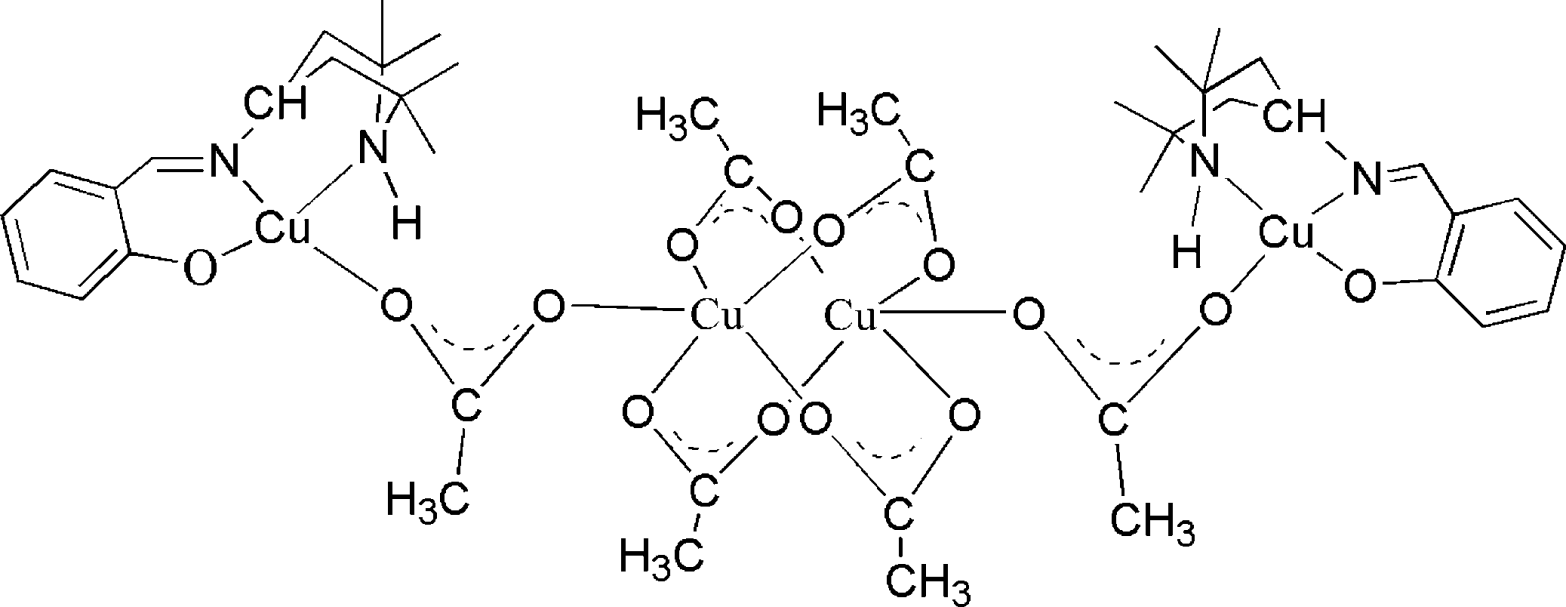


## Structural commentary   

**Figure 1:**
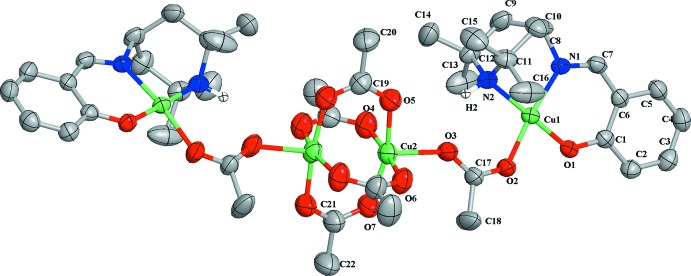
The mol­ecular structure of the title compound with 50% probability ellipsoids. For clarity, H atoms bonded to C atoms are not shown. The unlabeled part of the mol­ecule is related by the symmetry code (−*x* + 1, *y*, −*z* + 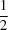).

The mol­ecular structure of the title complex is shown in Fig. 1 ▸. The complex lies across a twofold rotation axis. The asymmetric unit contains two independent Cu^II^ ions, Cu1 and Cu2. Cu1 is coordinated by atoms O1, N1 and N2 of a 2-{[(2,2,6,6-tetra­methyl­piperidin-4-yl)imino]­meth­yl}phenolate ligand and by atom O2 from an acetate group in a slightly distorted square-planar coordination environment. Cu2 is coordinated by atom O3 of the same acetate group mentioned above and by four bridging acetate ligands, which connect the asymmetric unit into a dimeric complex. Cu2 is in a distorted square-pyramidal coordination environment. The Cu⋯Cu distance is 2.6225 (9) Å. The piperidine rings are in boat conformations. Within the complex, there are two symmetry-equivalent intra­molecular N—H⋯O hydrogen bonds (Table 1 ▸).

## Supra­molecular features   

**Figure 2:**
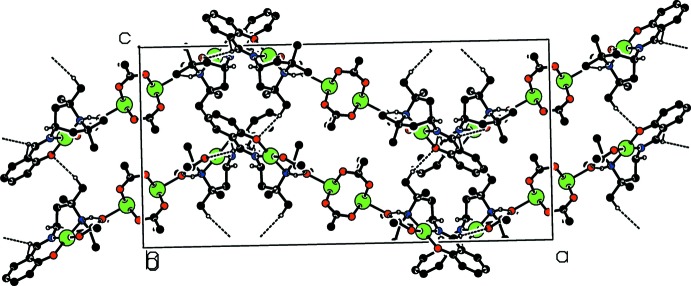
Part of the crystal structure, viewed along the *b* axis, with hydrogen bonds shown as dashed lines. Only H atoms involved in hydrogen bonding are shown.

In the crystal, weak C—H⋯O hydrogen bonds link the complex mol­ecules, forming a three-dimensional network (see Table 1 ▸ and Figs. 2 ▸ and 3 ▸).

## Database survey   

A search of the Cambridge Structural Database (Version 5.37, update 1; Groom & Allen, 2014 ▸) for compounds containing the same Schiff base ligand as the title compound found only one hit, namely bis­[*N*-(2,2,6,6-tetra­methyl­piperidin-4-yl)salicylaldiminato]copper(II) (Golovina *et al.*, 1975 ▸). In this compound, the ligand acts as only an *N*,*O* donor with the –N–H group remaining non-coordinating, unlike in the title compound. However, the precision of the determined geometric parameters is not sufficient to make a meaningful comparison with the title compound. Although, in a closely related compound, namely, hexa­kis­(μ_2_-acetato)­bis­[1-(5-bromo­salicylaldimino)-3-(2-methyl­piperidino)­propane]­tetracopper (Chiari *et al.*, 1993 ▸), the Cu—O and Cu—N distances for each coordination center are in agreement. A comprehensive study of the compound tetra­kis­(μ_2_-acetato)­bis­(acetic acid)dicopper(II), which is the basic core of the title compound, has been carried out by Vives *et al.* (2003 ▸).

## Synthesis and crystallization   

All chemicals and solvents used in the synthesis were analytical grade and used without further purification. A mixture of Cu(CH_3_COO)_2_·6H_2_O (12mg, 0.06 mmol) and SL ([2-{[(2,2,6,6-tetramethylpiperidin-4-yl)imino]methyl}phenolate]) (13 mg, 0.05 mmol) was treated in MeOH solvent (4 mL) under ultrasonic irradiation at ambient temperature to give a clear solution. The resultant solution was allowed to evaporate slowly in darkness at ambient temperature for several days to give blue crystals suitable for X-ray diffraction.

## Refinement   

Crystal data, data collection and structure refinement details are summarized in Table 2 ▸. Hydrogen atoms were placed in calculated positions with C—H = 0.94–0.99, N—H = 0.92 Å and were included in a riding-motion approximation with *U*_iso_(H) = 1.2*U*_eq_(C,N) or 1.5*U*_eq_(C_meth­yl_).

## Supplementary Material

Crystal structure: contains datablock(s) I. DOI: 10.1107/S2056989016005041/lh5808sup1.cif

Structure factors: contains datablock(s) I. DOI: 10.1107/S2056989016005041/lh5808Isup2.hkl

CCDC reference: 1470356

Additional supporting information:  crystallographic information; 3D view; checkCIF report

## Figures and Tables

**Figure 3:**
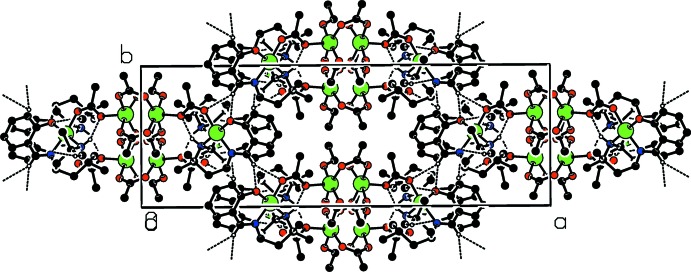
Part of the crystal structure, viewed along the *c* axis, with hydrogen bonds shown as dashed lines. Only H atoms involved in hydrogen bonding are shown.

**Table 1:** Hydrogen-bond geometry (Å, °)

*D*—H⋯*A*	*D*—H	H⋯*A*	*D*⋯*A*	*D*—H⋯*A*
N2—H2⋯O3	0.92	1.96	2.789 (3)	149
C7—H7⋯O1^i^	0.94	2.27	3.026 (3)	137
C7—H7⋯O2^i^	0.94	2.59	3.460 (3)	153
C15—H15*B*⋯O1^ii^	0.97	2.54	3.490 (4)	165

Symmetry codes: (i) 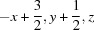; (ii) 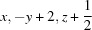.

**Table 2:** Experimental details

Crystal data	
Chemical formula	[Cu_4_(C_2_H_3_O_2_)_6_(C_16_H_23_N_2_O)_2_]
*M*_r_	1127.19
Crystal system, space group	Orthorhombic, *P* *b* *c* *n*
Temperature (K)	250
*a*, *b*, *c* (Å)	31.2431 (6), 10.7872 (2), 15.2556 (3)
*V* (Å^3^)	5141.53 (18)
*Z*	4
Radiation type	Cu *K*α
μ (mm^−1^)	2.40
Crystal size (mm)	0.10 × 0.10 × 0.05

Data collection	
Diffractometer	Agilent Gemini S Ultra CCD
Absorption correction	Multi-scan (*CrysAlis PRO*; Agilent, 2014 ▸)
*T*_min_, *T*_max_	0.718, 1.000
No. of measured, independent and observed [*I* > 2σ(*I*)] reflections	12793, 5096, 3794
*R*_int_	0.025
(sin θ/λ)_max_ (Å^−1^)	0.623

Refinement	
*R*[*F* ^2^ > 2σ(*F* ^2^)], *wR*(*F* ^2^), *S*	0.038, 0.109, 1.05
No. of reflections	5096
No. of parameters	305
H-atom treatment	H-atom parameters constrained
Δρ_max_, Δρ_min_ (e Å^−3^)	0.24, −0.43

Computer programs: *CrysAlis PRO* (Agilent, 2014 ▸), *SHELXS97* (Sheldrick, 2008 ▸), *SHELXL97* (Sheldrick, 2008 ▸), *OLEX2* (Dolomanov *et al.*, 2009 ▸) and *PLATON* (Spek, 2009 ▸), *OLEX2* (Dolomanov *et al.*, 2009 ▸).

## supplementary crystallographic information

### Crystal data

**Table:**

[Cu_4_(C_2_H_3_O_2_)_6_(C_16_H_23_N_2_O)_2_]	*D*_x_ = 1.456 Mg m^−^^3^
*M**_r_* = 1127.19	Cu *K*α radiation, λ = 1.54178 Å
Orthorhombic, *P**b**c**n*	Cell parameters from 4275 reflections
*a* = 31.2431 (6) Å	θ = 5.2–73.9°
*b* = 10.7872 (2) Å	µ = 2.40 mm^−^^1^
*c* = 15.2556 (3) Å	*T* = 250 K
*V* = 5141.53 (18) Å^3^	Block, blue
*Z* = 4	0.1 × 0.1 × 0.05 mm
*F*(000) = 2336	

### Data collection

**Table:**

Agilent Gemini S Ultra CCD diffractometer	5096 independent reflections
Radiation source: fine-focus sealed tube	3794 reflections with *I* > 2σ(*I*)
Graphite monochromator	*R*_int_ = 0.025
φ and ω scans	θ_max_ = 74.0°, θ_min_ = 4.3°
Absorption correction: multi-scan (*CrysAlis PRO*; Agilent, 2014)	*h* = −37→38
*T*_min_ = 0.718, *T*_max_ = 1.000	*k* = −12→13
12793 measured reflections	*l* = −18→12

### Refinement

**Table:**

Refinement on *F*^2^	Primary atom site location: structure-invariant direct methods
Least-squares matrix: full	Secondary atom site location: difference Fourier map
*R*[*F*^2^ > 2σ(*F*^2^)] = 0.038	Hydrogen site location: inferred from neighbouring sites
*wR*(*F*^2^) = 0.109	H-atom parameters constrained
*S* = 1.05	*w* = 1/[σ^2^(*F*_o_^2^) + (0.0512*P*)^2^ + 2.024*P*] where *P* = (*F*_o_^2^ + 2*F*_c_^2^)/3
5096 reflections	(Δ/σ)_max_ = 0.002
305 parameters	Δρ_max_ = 0.24 e Å^−^^3^
0 restraints	Δρ_min_ = −0.43 e Å^−^^3^

### Special details

**Table:**

Geometry. All e.s.d.'s (except the e.s.d. in the dihedral angle between two l.s. planes) are estimated using the full covariance matrix. The cell e.s.d.'s are taken into account individually in the estimation of e.s.d.'s in distances, angles and torsion angles; correlations between e.s.d.'s in cell parameters are only used when they are defined by crystal symmetry. An approximate (isotropic) treatment of cell e.s.d.'s is used for estimating e.s.d.'s involving l.s. planes.
Refinement. Refinement of *F*^2^ against ALL reflections. The weighted *R*-factor *wR* and goodness of fit *S* are based on *F*^2^, conventional *R*-factors *R* are based on *F*, with *F* set to zero for negative *F*^2^. The threshold expression of *F*^2^ > σ(*F*^2^) is used only for calculating *R*-factors(gt) *etc*. and is not relevant to the choice of reflections for refinement. *R*-factors based on *F*^2^ are statistically about twice as large as those based on *F*, and *R*- factors based on ALL data will be even larger.

### Fractional atomic coordinates and isotropic or equivalent isotropic displacement parameters (Å^2^)

**Table:**

	*x*	*y*	*z*	*U*_iso_*/*U*_eq_	
Cu1	0.684926 (12)	0.97594 (3)	0.05439 (3)	0.03840 (12)	
Cu2	0.537735 (15)	0.84119 (4)	0.21238 (3)	0.05264 (14)	
O1	0.71888 (6)	0.89442 (17)	−0.03139 (13)	0.0428 (4)	
N1	0.72175 (7)	1.1220 (2)	0.05451 (16)	0.0419 (5)	
O2	0.65473 (7)	0.81724 (18)	0.06203 (15)	0.0551 (6)	
O3	0.59817 (7)	0.85329 (19)	0.14558 (16)	0.0592 (6)	
O5	0.55006 (8)	0.9679 (2)	0.30302 (16)	0.0652 (6)	
N2	0.64737 (7)	1.0688 (2)	0.13950 (15)	0.0410 (5)	
H2	0.6248	1.0176	0.1529	0.049*	
O4	0.51510 (8)	0.9730 (2)	0.13830 (16)	0.0665 (6)	
C6	0.77655 (9)	1.0411 (2)	−0.04343 (18)	0.0398 (6)	
C7	0.75839 (9)	1.1298 (2)	0.0151 (2)	0.0444 (6)	
H7	0.7747	1.2014	0.0263	0.053*	
O7	0.50945 (8)	0.7157 (2)	0.13683 (17)	0.0713 (7)	
O6	0.55359 (8)	0.7104 (2)	0.29423 (17)	0.0677 (7)	
C2	0.77766 (9)	0.8474 (3)	−0.12210 (18)	0.0447 (6)	
H2A	0.7654	0.7700	−0.1355	0.054*	
C12	0.62916 (10)	1.1791 (3)	0.0926 (2)	0.0487 (7)	
C5	0.81565 (9)	1.0710 (3)	−0.0834 (2)	0.0501 (7)	
H5	0.8288	1.1469	−0.0698	0.060*	
C4	0.83518 (10)	0.9928 (3)	−0.1415 (2)	0.0545 (8)	
H4	0.8611	1.0152	−0.1686	0.065*	
C19	0.52267 (12)	1.0102 (3)	0.3548 (2)	0.0578 (8)	
C17	0.62166 (10)	0.7826 (3)	0.1042 (2)	0.0491 (7)	
C1	0.75614 (8)	0.9269 (2)	−0.06385 (17)	0.0385 (6)	
C21	0.47211 (12)	0.6744 (3)	0.1491 (2)	0.0622 (9)	
C11	0.67169 (11)	1.0900 (3)	0.2232 (2)	0.0507 (7)	
C8	0.70868 (11)	1.2271 (3)	0.1102 (2)	0.0518 (8)	
H8	0.7300	1.2945	0.1042	0.062*	
C20	0.53536 (14)	1.1180 (4)	0.4132 (3)	0.0764 (11)	
H20A	0.5659	1.1330	0.4078	0.115*	
H20B	0.5286	1.0983	0.4737	0.115*	
H20C	0.5198	1.1916	0.3956	0.115*	
C15	0.64393 (14)	1.1303 (4)	0.2999 (2)	0.0746 (11)	
H15A	0.6317	1.2110	0.2875	0.112*	
H15B	0.6613	1.1352	0.3525	0.112*	
H15C	0.6211	1.0705	0.3085	0.112*	
C3	0.81596 (10)	0.8793 (3)	−0.1599 (2)	0.0520 (7)	
H3	0.8294	0.8239	−0.1987	0.062*	
C13	0.61459 (16)	1.1326 (4)	0.0033 (3)	0.0863 (14)	
H13A	0.6390	1.1004	−0.0287	0.129*	
H13B	0.6019	1.2004	−0.0293	0.129*	
H13C	0.5936	1.0673	0.0110	0.129*	
C16	0.69203 (16)	0.9646 (3)	0.2464 (3)	0.0882 (15)	
H16A	0.6698	0.9024	0.2519	0.132*	
H16B	0.7074	0.9718	0.3014	0.132*	
H16C	0.7118	0.9405	0.2004	0.132*	
C10	0.70695 (12)	1.1862 (3)	0.2050 (2)	0.0604 (9)	
H10A	0.7021	1.2590	0.2421	0.073*	
H10B	0.7347	1.1507	0.2212	0.073*	
C18	0.61159 (14)	0.6453 (3)	0.1017 (3)	0.0853 (14)	
H18A	0.5920	0.6285	0.0539	0.128*	
H18B	0.5985	0.6207	0.1567	0.128*	
H18C	0.6378	0.5988	0.0929	0.128*	
C9	0.66490 (11)	1.2754 (3)	0.0824 (2)	0.0597 (9)	
H9A	0.6663	1.3016	0.0210	0.072*	
H9B	0.6578	1.3485	0.1177	0.072*	
C14	0.59022 (12)	1.2356 (3)	0.1380 (3)	0.0743 (11)	
H14A	0.5704	1.1702	0.1542	0.111*	
H14B	0.5762	1.2930	0.0984	0.111*	
H14C	0.5993	1.2795	0.1903	0.111*	
C22	0.45822 (14)	0.5698 (4)	0.0893 (3)	0.0949 (15)	
H22A	0.4715	0.5800	0.0322	0.142*	
H22B	0.4273	0.5712	0.0829	0.142*	
H22C	0.4670	0.4911	0.1144	0.142*	

### Atomic displacement parameters (Å^2^)

**Table:**

	*U*^11^	*U*^22^	*U*^33^	*U*^12^	*U*^13^	*U*^23^
Cu1	0.0381 (2)	0.0315 (2)	0.0456 (2)	−0.00219 (16)	0.00017 (17)	−0.00167 (16)
Cu2	0.0483 (3)	0.0563 (3)	0.0533 (3)	−0.0039 (2)	0.0161 (2)	−0.0029 (2)
O1	0.0406 (10)	0.0352 (9)	0.0526 (11)	−0.0049 (8)	0.0037 (9)	−0.0039 (8)
N1	0.0436 (12)	0.0312 (11)	0.0509 (13)	−0.0015 (10)	−0.0007 (11)	−0.0017 (10)
O2	0.0482 (11)	0.0408 (11)	0.0763 (15)	−0.0090 (9)	0.0235 (11)	−0.0121 (10)
O3	0.0553 (13)	0.0473 (12)	0.0752 (15)	−0.0109 (10)	0.0275 (12)	−0.0134 (11)
O5	0.0609 (14)	0.0704 (15)	0.0645 (14)	−0.0060 (12)	0.0162 (12)	−0.0173 (12)
N2	0.0447 (12)	0.0349 (11)	0.0436 (12)	−0.0033 (10)	−0.0003 (10)	−0.0008 (10)
O4	0.0607 (15)	0.0753 (16)	0.0634 (14)	0.0009 (12)	0.0160 (12)	0.0144 (12)
C6	0.0365 (13)	0.0383 (14)	0.0444 (14)	0.0005 (11)	−0.0040 (11)	0.0067 (11)
C7	0.0458 (16)	0.0327 (13)	0.0546 (16)	−0.0046 (12)	−0.0058 (13)	0.0029 (12)
O7	0.0599 (14)	0.0801 (17)	0.0738 (16)	−0.0107 (13)	0.0179 (13)	−0.0235 (13)
O6	0.0575 (14)	0.0693 (15)	0.0763 (16)	0.0094 (12)	0.0200 (12)	0.0133 (13)
C2	0.0485 (16)	0.0435 (15)	0.0421 (14)	−0.0018 (13)	−0.0002 (13)	−0.0025 (12)
C12	0.0517 (17)	0.0435 (16)	0.0508 (16)	0.0091 (13)	−0.0024 (14)	−0.0015 (13)
C5	0.0420 (15)	0.0479 (16)	0.0604 (17)	−0.0067 (13)	−0.0029 (14)	0.0053 (14)
C4	0.0404 (16)	0.063 (2)	0.0597 (18)	−0.0049 (15)	0.0061 (14)	0.0055 (16)
C19	0.064 (2)	0.0598 (19)	0.0500 (17)	0.0040 (17)	0.0046 (16)	0.0012 (15)
C17	0.0456 (16)	0.0427 (15)	0.0590 (18)	−0.0077 (13)	0.0100 (15)	−0.0057 (14)
C1	0.0392 (13)	0.0367 (13)	0.0397 (13)	0.0001 (11)	−0.0047 (11)	0.0062 (11)
C21	0.058 (2)	0.062 (2)	0.066 (2)	−0.0063 (17)	0.0101 (17)	−0.0064 (17)
C11	0.0637 (19)	0.0457 (16)	0.0428 (15)	−0.0044 (15)	−0.0083 (14)	−0.0011 (13)
C8	0.0570 (18)	0.0350 (14)	0.0635 (19)	−0.0107 (13)	0.0066 (15)	−0.0096 (13)
C20	0.082 (3)	0.081 (3)	0.066 (2)	0.001 (2)	0.003 (2)	−0.023 (2)
C15	0.091 (3)	0.085 (3)	0.0481 (18)	−0.024 (2)	0.0074 (19)	−0.0137 (18)
C3	0.0524 (17)	0.0567 (18)	0.0470 (16)	0.0070 (15)	0.0061 (14)	0.0001 (14)
C13	0.121 (4)	0.065 (2)	0.073 (3)	0.029 (2)	−0.045 (3)	−0.0074 (19)
C16	0.136 (4)	0.053 (2)	0.076 (3)	0.007 (2)	−0.053 (3)	0.0001 (19)
C10	0.064 (2)	0.060 (2)	0.0573 (19)	−0.0151 (17)	−0.0029 (16)	−0.0133 (16)
C18	0.081 (3)	0.0451 (19)	0.130 (4)	−0.0187 (19)	0.047 (3)	−0.016 (2)
C9	0.066 (2)	0.0383 (16)	0.075 (2)	0.0073 (15)	0.0198 (18)	0.0052 (15)
C14	0.056 (2)	0.060 (2)	0.106 (3)	0.0113 (18)	0.015 (2)	0.003 (2)
C22	0.078 (3)	0.093 (3)	0.114 (4)	−0.021 (2)	0.012 (3)	−0.041 (3)

### Geometric parameters (Å, º)

**Table:**

Cu1—O1	1.9004 (19)	C19—C20	1.518 (5)
Cu1—N1	1.951 (2)	C17—C18	1.515 (4)
Cu1—O2	1.958 (2)	C21—O6^i^	1.242 (4)
Cu1—N2	2.017 (2)	C21—C22	1.515 (5)
Cu2—Cu2^i^	2.6225 (9)	C11—C15	1.520 (5)
Cu2—O3	2.150 (2)	C11—C16	1.535 (5)
Cu2—O5	1.982 (2)	C11—C10	1.539 (4)
Cu2—O4	1.949 (2)	C8—H8	0.9900
Cu2—O7	1.986 (2)	C8—C10	1.513 (5)
Cu2—O6	1.948 (2)	C8—C9	1.524 (5)
O1—C1	1.313 (3)	C20—H20A	0.9700
N1—C7	1.295 (4)	C20—H20B	0.9700
N1—C8	1.474 (4)	C20—H20C	0.9700
O2—C17	1.273 (3)	C15—H15A	0.9700
O3—C17	1.232 (4)	C15—H15B	0.9700
O5—C19	1.251 (4)	C15—H15C	0.9700
N2—H2	0.9200	C3—H3	0.9400
N2—C12	1.500 (4)	C13—H13A	0.9700
N2—C11	1.503 (4)	C13—H13B	0.9700
O4—C19^i^	1.251 (4)	C13—H13C	0.9700
C6—C7	1.427 (4)	C16—H16A	0.9700
C6—C5	1.403 (4)	C16—H16B	0.9700
C6—C1	1.421 (4)	C16—H16C	0.9700
C7—H7	0.9400	C10—H10A	0.9800
O7—C21	1.262 (4)	C10—H10B	0.9800
O6—C21^i^	1.242 (4)	C18—H18A	0.9700
C2—H2A	0.9400	C18—H18B	0.9700
C2—C1	1.406 (4)	C18—H18C	0.9700
C2—C3	1.372 (4)	C9—H9A	0.9800
C12—C13	1.522 (5)	C9—H9B	0.9800
C12—C9	1.533 (5)	C14—H14A	0.9700
C12—C14	1.527 (4)	C14—H14B	0.9700
C5—H5	0.9400	C14—H14C	0.9700
C5—C4	1.368 (5)	C22—H22A	0.9700
C4—H4	0.9400	C22—H22B	0.9700
C4—C3	1.392 (4)	C22—H22C	0.9700
C19—O4^i^	1.251 (4)		
			
O1—Cu1—N1	92.58 (9)	N2—C11—C15	114.1 (3)
O1—Cu1—O2	84.57 (8)	N2—C11—C16	105.7 (2)
O1—Cu1—N2	176.50 (9)	N2—C11—C10	108.1 (2)
N1—Cu1—O2	171.93 (10)	C15—C11—C16	108.1 (3)
N1—Cu1—N2	86.64 (9)	C15—C11—C10	110.7 (3)
O2—Cu1—N2	96.65 (9)	C16—C11—C10	109.8 (3)
O3—Cu2—Cu2^i^	175.77 (7)	N1—C8—H8	109.0
O5—Cu2—Cu2^i^	82.49 (7)	N1—C8—C10	109.7 (3)
O5—Cu2—O3	96.79 (9)	N1—C8—C9	110.6 (3)
O5—Cu2—O7	164.08 (10)	C10—C8—H8	109.0
O4—Cu2—Cu2^i^	85.84 (7)	C10—C8—C9	109.5 (3)
O4—Cu2—O3	89.98 (10)	C9—C8—H8	109.0
O4—Cu2—O5	88.41 (11)	C19—C20—H20A	109.5
O4—Cu2—O7	89.96 (12)	C19—C20—H20B	109.5
O7—Cu2—Cu2^i^	81.60 (7)	C19—C20—H20C	109.5
O7—Cu2—O3	99.04 (9)	H20A—C20—H20B	109.5
O6—Cu2—Cu2^i^	87.02 (7)	H20A—C20—H20C	109.5
O6—Cu2—O3	97.15 (10)	H20B—C20—H20C	109.5
O6—Cu2—O5	90.16 (11)	C11—C15—H15A	109.5
O6—Cu2—O4	172.85 (10)	C11—C15—H15B	109.5
O6—Cu2—O7	89.50 (12)	C11—C15—H15C	109.5
C1—O1—Cu1	129.15 (17)	H15A—C15—H15B	109.5
C7—N1—Cu1	125.01 (19)	H15A—C15—H15C	109.5
C7—N1—C8	117.5 (2)	H15B—C15—H15C	109.5
C8—N1—Cu1	117.26 (19)	C2—C3—C4	120.8 (3)
C17—O2—Cu1	132.64 (19)	C2—C3—H3	119.6
C17—O3—Cu2	136.77 (19)	C4—C3—H3	119.6
C19—O5—Cu2	124.0 (2)	C12—C13—H13A	109.5
Cu1—N2—H2	106.9	C12—C13—H13B	109.5
C12—N2—Cu1	107.93 (17)	C12—C13—H13C	109.5
C12—N2—H2	106.9	H13A—C13—H13B	109.5
C11—N2—Cu1	109.16 (18)	H13A—C13—H13C	109.5
C11—N2—H2	106.9	H13B—C13—H13C	109.5
C11—N2—C12	118.5 (2)	C11—C16—H16A	109.5
C19^i^—O4—Cu2	121.9 (2)	C11—C16—H16B	109.5
C5—C6—C7	117.6 (3)	C11—C16—H16C	109.5
C5—C6—C1	119.7 (3)	H16A—C16—H16B	109.5
C1—C6—C7	122.7 (3)	H16A—C16—H16C	109.5
N1—C7—C6	126.7 (3)	H16B—C16—H16C	109.5
N1—C7—H7	116.6	C11—C10—H10A	108.9
C6—C7—H7	116.6	C11—C10—H10B	108.9
C21—O7—Cu2	124.5 (2)	C8—C10—C11	113.2 (3)
C21^i^—O6—Cu2	120.5 (2)	C8—C10—H10A	108.9
C1—C2—H2A	119.0	C8—C10—H10B	108.9
C3—C2—H2A	119.0	H10A—C10—H10B	107.7
C3—C2—C1	122.0 (3)	C17—C18—H18A	109.5
N2—C12—C13	106.2 (2)	C17—C18—H18B	109.5
N2—C12—C9	108.0 (2)	C17—C18—H18C	109.5
N2—C12—C14	113.7 (3)	H18A—C18—H18B	109.5
C13—C12—C9	110.5 (3)	H18A—C18—H18C	109.5
C13—C12—C14	107.4 (3)	H18B—C18—H18C	109.5
C14—C12—C9	110.8 (3)	C12—C9—H9A	108.9
C6—C5—H5	119.1	C12—C9—H9B	108.9
C4—C5—C6	121.9 (3)	C8—C9—C12	113.2 (3)
C4—C5—H5	119.1	C8—C9—H9A	108.9
C5—C4—H4	120.6	C8—C9—H9B	108.9
C5—C4—C3	118.7 (3)	H9A—C9—H9B	107.8
C3—C4—H4	120.6	C12—C14—H14A	109.5
O5—C19—C20	118.1 (3)	C12—C14—H14B	109.5
O4^i^—C19—O5	125.5 (3)	C12—C14—H14C	109.5
O4^i^—C19—C20	116.3 (3)	H14A—C14—H14B	109.5
O2—C17—C18	116.3 (3)	H14A—C14—H14C	109.5
O3—C17—O2	124.1 (3)	H14B—C14—H14C	109.5
O3—C17—C18	119.6 (3)	C21—C22—H22A	109.5
O1—C1—C6	123.1 (2)	C21—C22—H22B	109.5
O1—C1—C2	120.0 (2)	C21—C22—H22C	109.5
C2—C1—C6	116.9 (2)	H22A—C22—H22B	109.5
O7—C21—C22	116.0 (3)	H22A—C22—H22C	109.5
O6^i^—C21—O7	126.2 (3)	H22B—C22—H22C	109.5
O6^i^—C21—C22	117.8 (3)		
			
Cu1—O1—C1—C6	−5.6 (4)	O5—Cu2—O6—C21^i^	80.2 (3)
Cu1—O1—C1—C2	174.72 (19)	N2—Cu1—O1—C1	85.2 (15)
Cu1—N1—C7—C6	7.8 (4)	N2—Cu1—N1—C7	174.4 (3)
Cu1—N1—C8—C10	60.7 (3)	N2—Cu1—N1—C8	0.2 (2)
Cu1—N1—C8—C9	−60.1 (3)	N2—Cu1—O2—C17	1.7 (3)
Cu1—O2—C17—O3	7.8 (5)	N2—C12—C9—C8	7.7 (4)
Cu1—O2—C17—C18	−172.3 (3)	N2—C11—C10—C8	−2.6 (4)
Cu1—N2—C12—C13	44.4 (3)	O4—Cu2—O3—C17	−122.8 (3)
Cu1—N2—C12—C9	−74.1 (3)	O4—Cu2—O5—C19	80.8 (3)
Cu1—N2—C12—C14	162.4 (2)	O4—Cu2—O7—C21	−90.6 (3)
Cu1—N2—C11—C15	−165.8 (2)	O4—Cu2—O6—C21^i^	1.8 (11)
Cu1—N2—C11—C16	−47.1 (3)	C6—C5—C4—C3	−1.3 (5)
Cu1—N2—C11—C10	70.5 (3)	C7—N1—C8—C10	−113.9 (3)
Cu2^i^—Cu2—O3—C17	−131.2 (8)	C7—N1—C8—C9	125.2 (3)
Cu2^i^—Cu2—O5—C19	−5.2 (3)	C7—C6—C5—C4	−178.9 (3)
Cu2^i^—Cu2—O4—C19^i^	−1.9 (3)	C7—C6—C1—O1	0.7 (4)
Cu2^i^—Cu2—O7—C21	−4.8 (3)	C7—C6—C1—C2	−179.6 (3)
Cu2^i^—Cu2—O6—C21^i^	−2.2 (3)	O7—Cu2—O3—C17	−32.8 (4)
Cu2—O3—C17—O2	172.0 (2)	O7—Cu2—O5—C19	−3.5 (6)
Cu2—O3—C17—C18	−7.9 (6)	O7—Cu2—O4—C19^i^	79.7 (3)
Cu2—O5—C19—O4^i^	5.8 (5)	O7—Cu2—O6—C21^i^	−83.8 (3)
Cu2—O5—C19—C20	−172.6 (3)	O6—Cu2—O3—C17	57.8 (4)
Cu2—O7—C21—O6^i^	4.9 (6)	O6—Cu2—O5—C19	−92.2 (3)
Cu2—O7—C21—C22	−174.1 (3)	O6—Cu2—O4—C19^i^	−5.9 (11)
O1—Cu1—N1—C7	−9.0 (2)	O6—Cu2—O7—C21	82.3 (3)
O1—Cu1—N1—C8	176.8 (2)	C12—N2—C11—C15	70.2 (4)
O1—Cu1—O2—C17	−175.0 (3)	C12—N2—C11—C16	−171.1 (3)
O1—Cu1—N2—C12	−11.9 (15)	C12—N2—C11—C10	−53.5 (3)
O1—Cu1—N2—C11	−141.9 (14)	C5—C6—C7—N1	176.2 (3)
N1—Cu1—O1—C1	8.2 (2)	C5—C6—C1—O1	−177.7 (3)
N1—Cu1—O2—C17	115.4 (7)	C5—C6—C1—C2	2.0 (4)
N1—Cu1—N2—C12	65.23 (18)	C5—C4—C3—C2	1.5 (5)
N1—Cu1—N2—C11	−64.75 (18)	C1—C6—C7—N1	−2.2 (5)
N1—C8—C10—C11	−66.2 (4)	C1—C6—C5—C4	−0.4 (4)
N1—C8—C9—C12	62.4 (4)	C1—C2—C3—C4	0.2 (5)
O2—Cu1—O1—C1	−164.2 (2)	C11—N2—C12—C13	169.0 (3)
O2—Cu1—N1—C7	60.1 (8)	C11—N2—C12—C9	50.5 (3)
O2—Cu1—N1—C8	−114.1 (7)	C11—N2—C12—C14	−73.0 (4)
O2—Cu1—N2—C12	−122.17 (18)	C8—N1—C7—C6	−178.0 (3)
O2—Cu1—N2—C11	107.85 (18)	C15—C11—C10—C8	−128.3 (3)
O3—Cu2—O5—C19	170.6 (3)	C3—C2—C1—O1	177.8 (3)
O3—Cu2—O4—C19^i^	178.8 (3)	C3—C2—C1—C6	−2.0 (4)
O3—Cu2—O7—C21	179.5 (3)	C13—C12—C9—C8	−108.1 (3)
O3—Cu2—O6—C21^i^	177.1 (3)	C16—C11—C10—C8	112.3 (3)
O5—Cu2—O3—C17	148.8 (3)	C10—C8—C9—C12	−58.6 (4)
O5—Cu2—O4—C19^i^	−84.4 (3)	C9—C8—C10—C11	55.3 (4)
O5—Cu2—O7—C21	−6.5 (6)	C14—C12—C9—C8	133.0 (3)

Symmetry code: (i) −*x*+1, *y*, −*z*+1/2.

### Hydrogen-bond geometry (Å, º)

**Table:**

*D*—H···*A*	*D*—H	H···*A*	*D*···*A*	*D*—H···*A*
N2—H2···O3	0.92	1.96	2.789 (3)	149
C7—H7···O1^ii^	0.94	2.27	3.026 (3)	137
C7—H7···O2^ii^	0.94	2.59	3.460 (3)	153
C15—H15*B*···O1^iii^	0.97	2.54	3.490 (4)	165

Symmetry codes: (ii) −*x*+3/2, *y*+1/2, *z*; (iii) *x*, −*y*+2, *z*+1/2.
